# Using Open Street Map Data in Environmental Exposure Assessment Studies: Eastern Massachusetts, Bern Region, and South Israel as a Case Study

**DOI:** 10.3390/ijerph15112443

**Published:** 2018-11-01

**Authors:** Itai Kloog, Lara Ifat Kaufman, Kees de Hoogh

**Affiliations:** 1 Department of Geography and Environmental Development, Ben-Gurion University of the Negev, Beer Sheva 8410501, Israel; laraifat@post.bgu.ac.il; 2 Environmental Exposure and Health Unit, Department of Epidemiology and Public Health, Swiss Tropical and Public Health Institute, Socinstrasse 57, 4051 Basel, Switzerland; c.dehoogh@unibas.ch; 3 Epidemiology and Public Health, Socinstrasse 57, Basel University of Basel, 4002 Basel, Switzerland

**Keywords:** OpenStreetMap, exposure assessment, completeness, positional accuracy, public health, epidemiology

## Abstract

There is an increase in the awareness of the importance of spatial data in epidemiology and exposure assessment (EA) studies. Most studies use governmental and ordnance surveys, which are often expensive and sparsely updated, while in most developing countries, there are often no official geo-spatial data sources. OpenStreetMap (OSM) is an open source Volunteered Geographic Information (VGI) mapping project. Yet very few environmental epidemiological and EA studies have used OSM as a source for road data. Since VGI data is either noncommercial or governmental, the validity of OSM is often questioned. We investigate the robustness and validity of OSM data for use in epidemiological and EA studies. We compared OSM and Governmental Major Road Data (GRD) in three different regions: Massachusetts, USA; Bern, Switzerland; and Beer-Sheva, South Israel. The comparison was done by calculating data completeness, positional accuracy, and EA using traditional exposure methods. We found that OSM data is fairly complete and accurate in all regions. The results in all regions were robust, with Massachusetts showing the best fits (R^2^ 0.93). Results in Bern (R^2^ 0.78) and Beer-Sheva (R^2^ 0.77) were only slightly lower. We conclude by suggesting that OSM data can be used reliably in environmental assessment studies.

## 1. Introduction

For the past 30 years, there is increasing awareness of the importance of spatial data in epidemiological studies [1]. Understanding the spatial distribution of hazards (i.e., air pollution measurements, transport networks, land cover, wind speed, etc.) and population data (location, socioeconomic status, education, etc.) is critical for exposure assessment studies. The Geographical Information System (GIS) allows combining all the necessary data for exposure assessment studies [2,3]. GIS data used in those studies are usually from known trusted sources, such as government or ordnance surveys, which are usually expensive and, in some locations, sparsely updated. In addition, in most developing countries, there are no official GIS data sources, making it more difficult to conduct exposure assessment studies [4]. In contrast, Volunteered Geographic Information (VGI) [5,6], i.e., OpenStreetMap, Google maps etc., are usually free of charge and frequently updated by users around the world [5].

OpenStreetMap (OSM) is an open source and open content license VGI mapping project that aims to create and provide free worldwide geographic data. Since it was founded in 2004, millions of users around the world have contributed voluntarily to the creation of global street maps using GPS, digitizing from free aerial images or other non-copyrights sources [7]. The number of people using and producing VGI is constantly increasing [8,9], since VGI data is usually the cheapest source of geographic data (data from OSM is free and open). As the number of contributors grow, the quality of OSM data improves, since users can update the data more frequently [10,11]. Moreover, people who contribute to VGI usually focus on areas most familiar to them. This local knowledge is one of the greatest benefits of VGI [12,13].

Although OSM has a large number of contributors, most of them do not have a professional background in GIS, and do not use professional equipment [5,10,12,14]. In addition, it has been shown that the number of users per area improves the overall quality of OSM, but not in a linear way [11]. Therefore, there are concerns about the quality and reliability of VGI as an information source, which have led many studies to assess the quality of OSM data [15]. Some recent studies focusing on this issue found that although most VGI users do not have GIS experience, the difference in mapping between expert and non-expert users was minor [16]. In addition, many studies assessed the quality of OSM data by comparing it to more reliable reference data, such as governmental data or ordnance surveys (OS), and calculated data completeness [11,17,18,19,20,21,22,23], positional accuracy (PA) [10,17,18,21,24,25], and thematic accuracy [11,22,26,27]. The studies found that completeness and positional accuracy improves over time, but vary in space; urban areas are more complete and accurate compared to rural areas [17,18,19,24,25]. In addition, there was a difference in the thematic accuracy of roads. For example, when comparing OSM and governmental data in France, major road classification showed a thematic accuracy of 100%, while for secondary roads, the thematic accuracy was 49% [11]. An important study by Mocnik and colleagues [28] discussed data quality measures for VGI at length. They present concepts of data quality measures by the source of information to which the data is compared to assess their quality. They use several examples of VGI, which also applies to other geographical data and data in general. They summarize by showing how this used information provides an alternative grounding of the data, which potentially refers to the environment in a different way than the original grounding. This also sheds light on the quality of the data evaluated in the context of OSM data. This grounding-based ontology can improve the understanding of how different data quality measures correlate, and how they can mutually complement each other. Another important part of VGI is the coordinated effort to create and maintain the data. The interpretation and analysis of VGI data is often only possible when considering the social process that leads to their creation. A holistic understanding of the OSM data and its creation process can only be gained by examining several datasets. Thus, a more complete understanding of which factors influence the emergence of the data is often still missing [29].

Although many studies have focused on OSM quality, studies from different fields focused on the use of OSM data for different purposes, such as: Urban planning [30,31], routing and navigation [32,33,34,35,36,37,38,39,40,41], transportation [42,43,44,45], mobility and accessibility [46,47,48], mapping and geocoding [49,50,51], and its use in disaster events [52,53]. Mobasheri and colleagues [40] presented a modified methodology based on data mining techniques for constructing sidewalk geometries using multiple GPS traces collected by wheelchair users during an urban travel experiment. They applied their methods to a case study in Heidelberg, Germany. The constructed sidewalk geometries were compared to the official dataset. They showed that the constructed sidewalk network overlays with 96% of the official reference dataset. In terms of positional accuracy, they present very accurate Root mean square error (RMSE) values of 0.93 m. Another study by Mobasheri and colleagues [41] looked at the assessment of sidewalk data in OSM. They present results of “awareness raising” using tools for tagging accessibility data into OSM database for enriching the sidewalk data completeness. They carried out several experiments in different European cities. They conclude that awareness raising and public engagement have a direct effect on the enrichment of data completeness, especially for those kinds of information that target special needs (e.g., sidewalk information). 

Some studies concluded that data from OSM is fairly complete and accurate in most regions and its quality is constantly improving [17,18,21,24]. Furthermore, studies have shown that OSM data can be used for urban planning and environmental noise pollution [49,50,51].

Despite these findings, very few environmental epidemiological and exposure assessment studies have used OSM as a source for road and spatial data (Table 1). Most of these studies [54,55,56,57,58,59,60,61] used original OSM data, without making any changes to the OSM data. A few studies, however, found that misclassification in the OSM road type affected the exposure assessment results, which they corrected by modifying the classification based on local knowledge [62,63]. Others found OSM encoding of road network data was not uniform, and they needed to use governmental road network instead of OSM [64]. Also, in several studies, OSM roads were divided into different categories, but no consistency was found in categories’ affiliation: For example, in the UK, motorway, primary, and trunk roads were classified as major roads [54]. In Greece, however, motorways were classified as a separate category [57].

Due to the scarce number of studies using OSM data in environmental epidemiology studies, there is a real need to investigate the robustness and validity of OSM data to be used in such studies. Therefore, the aim of this research is to test the viability of OSM data as a reliable and robust source for exposure assessment data used in environmental epidemiology studies. To assess this, we compared traffic-related air pollution exposure assessment results, calculated using both OSM and governmental/commercial road data, in three different regions of the world: North America (Eastern Massachusetts, USA), Europe (Bern Region, Switzerland), and the Middle East (south region, Israel). Firstly, we checked the validity of OSM versus governmental or commercial data in our study regions. Next, we evaluated the application of the data by comparing the use of both road datasets in exposure assessment studies in the different regions. To the best of our knowledge, we evaluate for the first-time the viability of using OSM data as a free, global, and readily available data source in environmental exposure assessment studies comparing both OSM and GRD data in the process. 

## 2. Materials and Methods

### 2.1. Research Area

**Figure 1:**
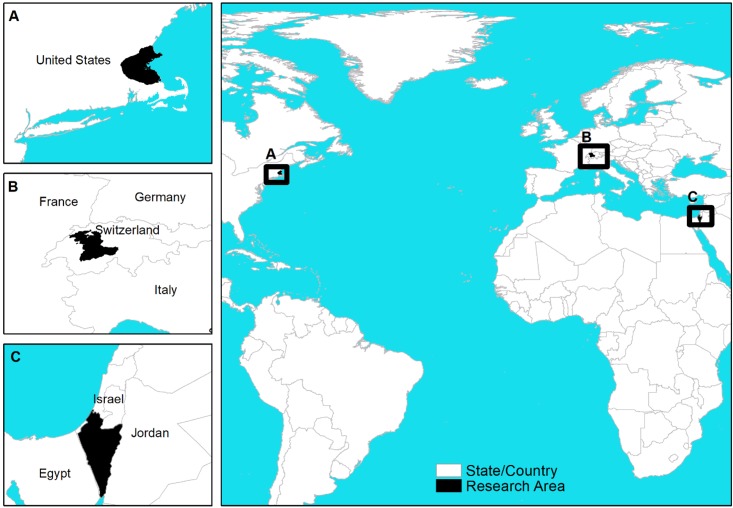
Research Area.

The research focused on three districts in developed countries: North America (Eastern Massachusetts, USA), Europe (Bern region, Switzerland), and the Middle East (south region, Israel) (Figure 1).

The Eastern Massachusetts study region, approximately 5000 km^2^ in size, is located in the north eastern United States. The region’s main city, Boston, is the economic and cultural hub of New England, and has over 617,000 residents [64].

The Bern region is located in East Switzerland with an approximate area of 6000 km^2^ and a population of 1,009,400 residents (about 168 people per square kilometer). Bern is the capital city of Switzerland, with approximately 125,000 inhabitants [65]. 

The southern region of Israel is about 14,500 km^2^, with its main city, Beer-Sheva, located in the north. The region has a population of 1,192,300 million distributed sparsely, with a low population density (84 people per square kilometer); in comparison, the average population density of Israel is 376 people per square kilometer [66].

### 2.2. Data Collection and Preparation

OSM road data was downloaded from the GeoFabrik Company, which specializes in working with OpenStreetMap. The data is updated daily and can be downloaded freely from their website [67]. Road data for the study areas were downloaded 8 November 2015.

Massachusetts governmental road and building data is accessible through the Massachusetts Office of Geographic Information [68] website. The road data is up-to-date until 31 December 2013. The data were released by MassGIS on 13 June 2014. The building data is updated to March 2016.

Swisstopo is the Federal Office of Topography in Switzerland, which produces GIS databases. VECTOR25 is one of their products that include road network data and building data. The latest version was released in 2008; this version was used in this research.

For southern Israel, we used the GIS vector layers from the 2011 road network from a survey company [69], and building data from Beer-Sheva municipality, updated August 2015.

OSM uses the World Geodetic 1984 (WGS84) System, while each country uses different projections to ensure a better representation of their topography, i.e., Survey of Israel (MAPA) uses Israel Transverse Mercator (ITM) projection. To compare between the different layers, each OSM layer was re-projected to the original projection of the governmental/survey layer.

Exposure assessment research traditionally uses density of and/or distance to major roads to assess the exposure to traffic related pollutants [70,71]. We thus compared the quality and exposure assessment results between OSM and GRD using major roads only. Major roads were defined as motorways, main roads of major importance, and other main roads, a commonly used road classification [72].

### 2.3. Assessing Data Quality

OSM road data quality was assessed by comparing it to the governmental (or survey) road data (GRD), calculating the completeness and positional accuracy in each area using the Haklay’s [17] method, also used in previous studies. A grid of 1 × 1 km^2^ covering the three study areas was created using the “Fishnet” tool in ArcGIS 10.3.1. The lengths of both road datasets were calculated in each cell using the “Sum Line Length” tool in QGIS version 2.16.2 to calculate OSM completeness percentage using the following calculation:

$$\;\mathrm{OSM}\;\mathrm{Completeness}\;\left(\%\right)=\frac{\mathrm{OSM}\;\mathrm{major}\;\mathrm{road}\;\mathrm{length}\;\left(m\right)}{\mathrm{major}\;\mathrm{GRD}\;\mathrm{length}\;\left(m\right)}\times 100\;$$

The methodology used to evaluate the positional accuracy of OSM data is based on Hunter and Goodchild [73]. Roads are represented as lines in GIS while in the real-world, roads have area. To calculate if the position of the ‘untrusted’ data source (i.e., OSM) is in the same position as the ‘trusted’ data source (i.e., GRD), we used buffers to determine which percentage of the area from OSM was inside the area of GRD. To measure this accuracy, we created buffers of 15 m and 20 m around the GRD data, and a 1m buffer around the OSM road data. If the OSM 1m buffer was inside the 15–20 m GRD buffer in the same grid—the positional accuracy of OSM would be 100% in this grid. In each cell of the grid, the total intersection area between the two buffers was divided by the total OSM buffer area inside the grid to measure the positional accuracy percentage:

$$\;\mathrm{OSM}\;\mathrm{Positional}\;\mathrm{Accuracy}\;\left(\%\right)=\;\frac{\mathrm{Intersected}\;\mathrm{area}\;\mathrm{between}\;\mathrm{OSM}\;\mathrm{buffer}\;\mathrm{and}\;\mathrm{GRD}\;\mathrm{buffer}\;\left(m^{2}\right)}{\mathrm{OSM}\;\mathrm{buffer}\;\mathrm{area}\;\left(m^{2}\right)}\times 100\;$$

### 2.4. Exposure Assessment Comparison

To evaluate OSM road data for exposure assessment research, we used commonly used exposure assessment methods metrics for characterizing exposure to traffic-related air pollutants as commonly performed in previous studies [70,71]. These metrics include: 1. The proximity (distance) to major roads- important since concentrations of traffic-related air pollutants rapidly decrease with distance from major roads; and 2. traffic (road) density-an important metric for all road air pollution density within study participants’ home addresses. We compared exposure assessment results from both commercial and OSM sources using a random sample of 10% of the buildings in each region (using the QGIS “Random Sampling” tool), representing a 10% random sample of the representative study population. A centroid was generated from each selected building polygon using the “Polygon Centroid” tool in Quantum Geographic Information System (QGIS).

#### 2.4.1. Distance to Nearest Major Road

Distance from the building centroid to the closest major road was calculated using the “arcpy.GenerateNearTable” Analysis. Using R version 3.3.0, a linear model was calculated to compare the OSM and GRD results. Adjusted R^2^ results of the linear regression allowed us to measure the percent of the difference/similarity between the results.

#### 2.4.2. Road Density

Road density was calculated using different sized buffers (50, 100, 200, 500, and 1000 m) from the building centroid, created using the Buffer tool in QGIS. In each buffer, the road length was calculated for major roads only, using the “Sum Line Length” tool in QGIS. Road density was calculated in R using the following calculation:

$$\;{\mathrm{Road}\;\mathrm{Density}\;\mathrm{in}\;x\;\mathrm{meters}\;\mathrm{buffer}\;}_{i\;\left(\mathrm{the}\;\mathrm{different}\;\mathrm{sized}\;\mathrm{buffers}\right)}=\frac{\mathrm{total}\;\mathrm{major}\;\mathrm{road}\;\mathrm{length}\;\mathrm{inside}\;\mathrm{the}\;\mathrm{buffer}\;\left(m\right)}{\mathrm{buffer}\;\mathrm{area}\;\left(m^{2}\right)}\;$$

A linear model was performed to compare between OSM and GRD results in the different buffers. Adjusted R^2^ results of the linear regression will again allow us to measure the percentage of the difference/similarity between the results.

## 3. Results

### 3.1. Descriptive Statistics

Descriptive statistics are shown in Table 2. Although the areas of the southern region in Israel (~14,500 km^2^) and Bern (~6000 km^2^) are bigger compared to the area of Eastern Massachusetts (~4900 km^2^), total road length is higher in Eastern Massachusetts, both for OSM and GRD.

### 3.2. Data Quality

#### 3.2.1. Completeness

**Figure 2:**
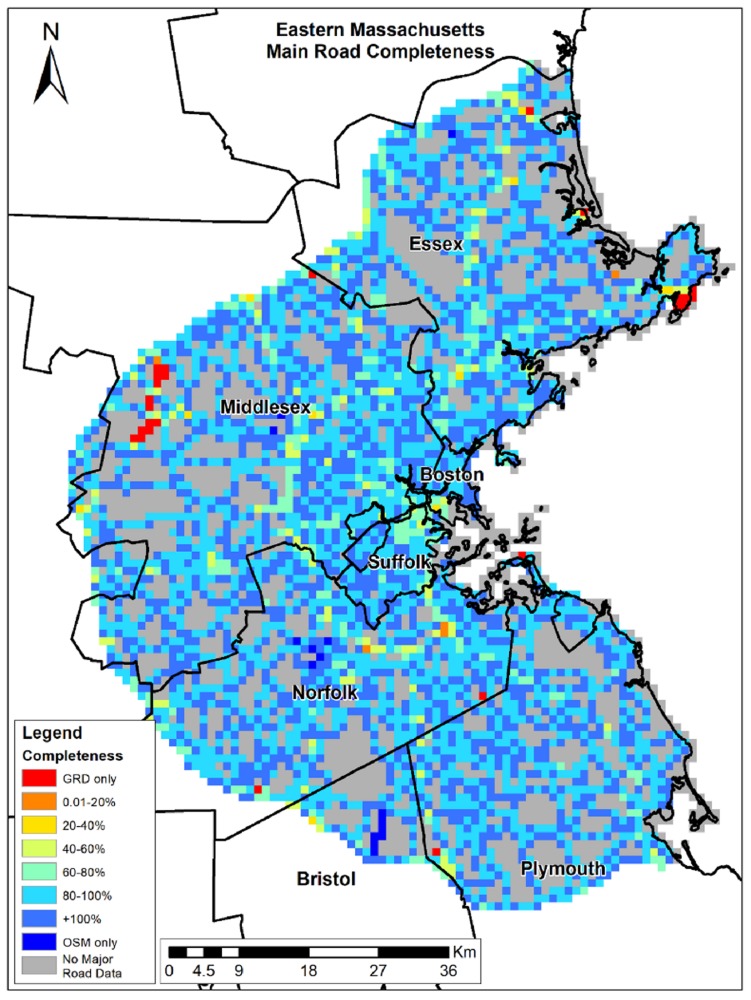
Eastern Massachusetts’ completeness (%).

**Figure 3:**
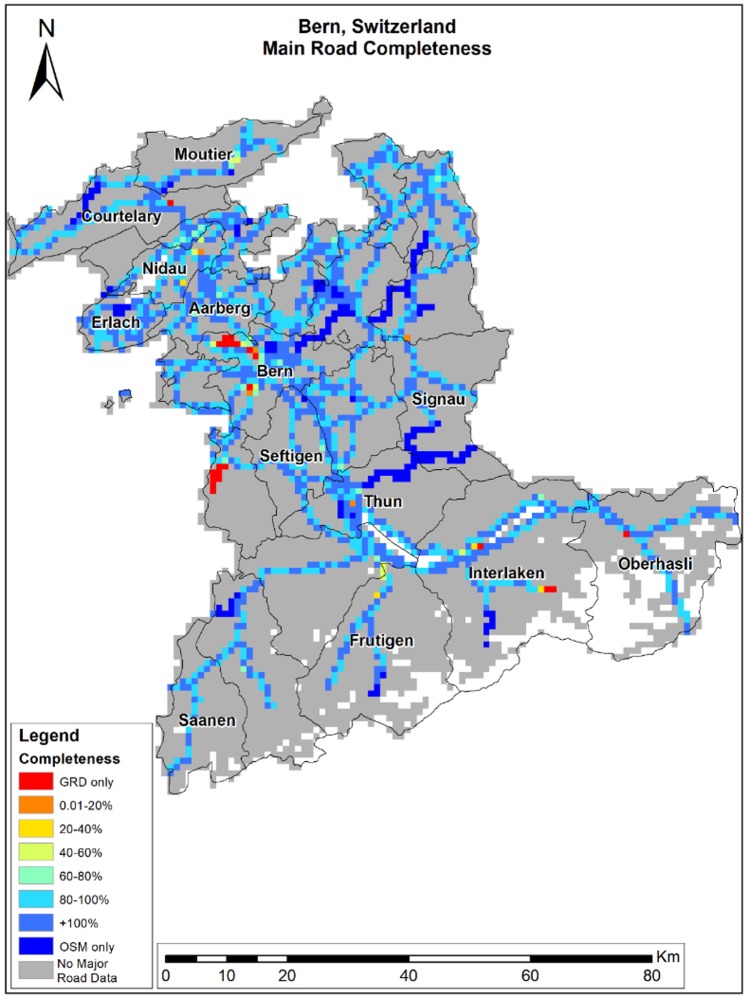
Bern region’s completeness (%).

**Figure 4:**
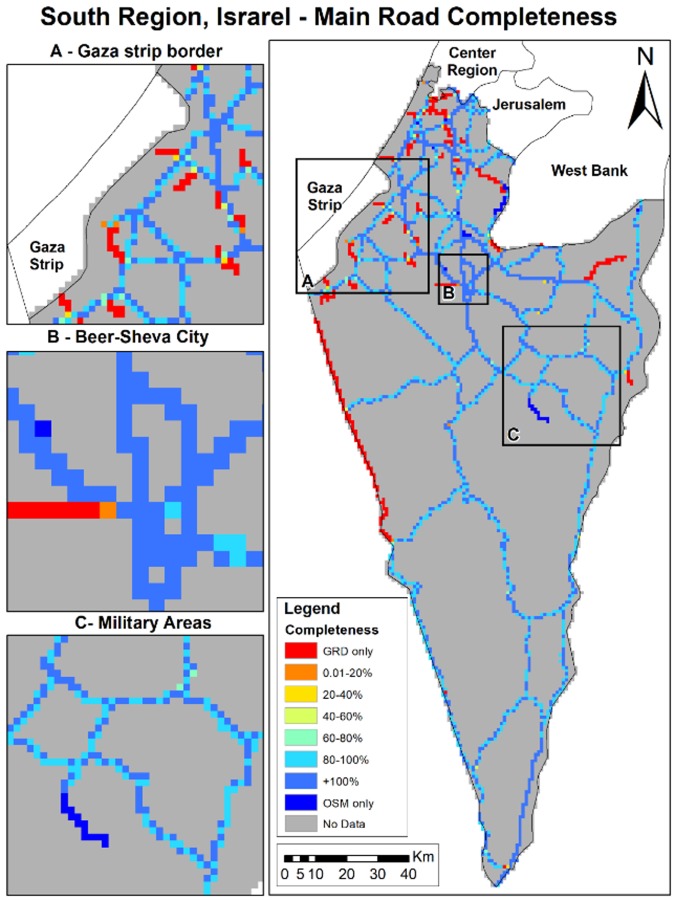
Israel’s south region’s completeness (%).

OSM completeness results vary across the different study areas (Table 3). In Eastern Massachusetts, average completeness is ~90%, while in Bern and the south region of Israel, OSM completeness is above 100%. Although the average completeness over all grid cells is very high, OSM completeness varies over space as shown in Figure 2, Figure 3 and Figure 4. As shown in Figure 2 and Figure 3, completeness percentages in Eastern Massachusetts and Bern are generally very high. In Israel’s southern region (Figure 4), completeness varies over space, and is low around the border regions and several areas in the center of the map (Figure 3A).

To compare completeness between rural areas and populated areas, we calculated the completeness for Boston, Bern city, and Beer-Sheva Metropolis city separately. We found that the completeness in Boston was ~6% lower compared to Eastern Massachusetts, and Bern city ~1% lower compared to Bern Region, but completeness in Beer-Sheva was ~62% higher than in the region. The areas colored in red are areas with a completeness of 0%, meaning GRD only. To understand whether the lower results are due to a lack of OSM roads or due to misclassification, we compared the original road data gathered from GeoFabrik, before filtering major roads only. We found that the lower result in Beer-Sheva city (Figure 3B) was not due to a lack of OSM data, but was caused by the difference in road class definitions; the same road was classified as a minor road in OSM (which was not included in the study area), while in GRD, it was defined as a regional highway. The slightly lower completeness results in Bern City (Figure 2) were also due to OSM misclassification; instead of primary or trunk classification, roads were classified as track, path, and residential roads, all of which were not included in major roads selection.

#### 3.2.2. Positional Accuracy

**Figure 5:**
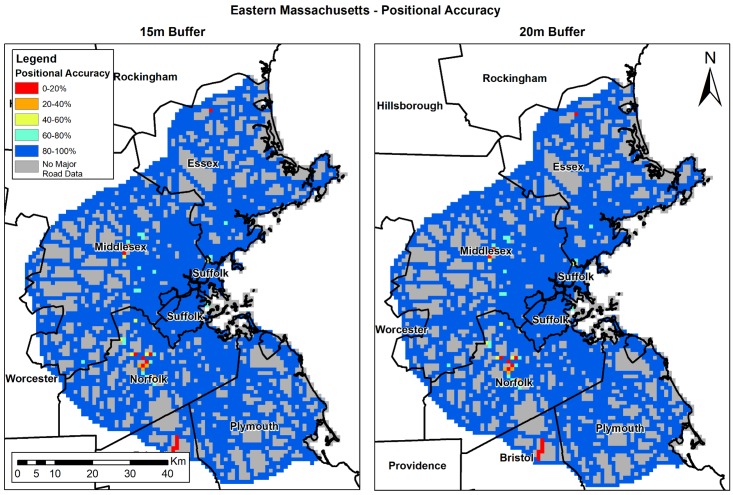
Eastern Massachusetts’ PA (%): 15 m (**left**) and 20 m (**right**) buffer.

**Figure 6:**
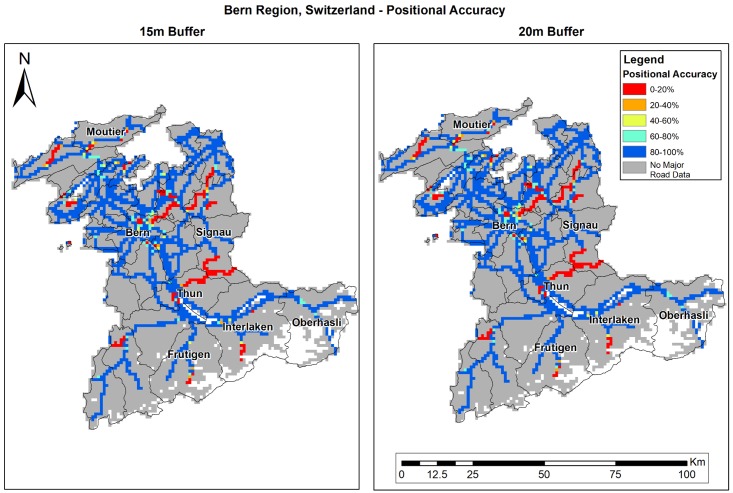
Bern region’s PA (%): 15 m (**left**) and 20 m (**right**) buffer.

**Figure 7:**
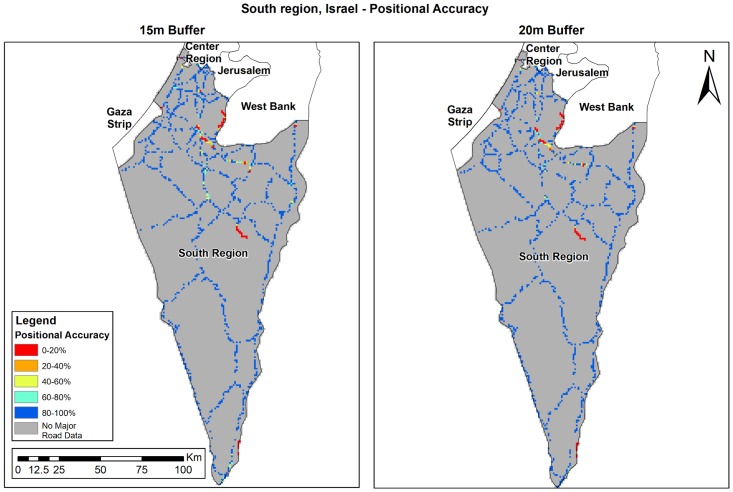
Israel’s south region’s PA (%): 15 m (**left**) and 20 m (**right**) buffer.

The positional accuracy (PA) analysis results show the current precision of OSM, and is calculated only in areas where OSM data exists. OSM PA varies in the different study areas (Table 4). In Eastern Massachusetts, PA is over 98%; in Israel’s southern region, PA is 94%; while in Bern, we found the lowest PA results (~88%). Results improved when the buffer size was increased in all study areas. Figure 5, Figure 6 and Figure 7 shows the results per study area. OSM PA varies over space, while in Eastern Massachusetts (Figure 5) and the south of Israel (Figure 7), PA is generally high; in Bern (Figure 6), the PA percent is very high in several areas (colored blue), but there are also several areas where PA is very low (colored red).

We also calculated the PA for Boston, Bern city, and Beer-Sheva Metropolis city separately (Table 4) to compare the total PA in rural areas with the PA in populated areas. We found that, compared to the whole region, PA in Boston was higher, while in Beer-Sheva, the results were lower compared to the whole region. In Bern, however, PA results were lower in the 15 m buffer and higher in the 20 m buffer. PA was only calculated in areas where OSM data exists and this could explain why some areas have a low PA; either the PA of OSM is actually low, or GRD is missing in those grid cells.

### 3.3. Exposure Assessment Comparison

The total number of buildings used and major roads selected can be found in Table 5. The number of buildings and major roads used in Eastern Massachusetts is higher than in the Bern region and Beer-Sheva city, since the total area of Eastern Massachusetts is significantly bigger (Table 2). There is a difference in major roads’ lengths between OSM and GRD in all study areas. There are three probable reasons for this difference: (1) The classification of major roads in OSM is affected by the user’s definition, thus it is possible that misclassification of roads affected the number of roads selected; (2) major roads are missing in OSM or GRD databases; and (3) a combination of the two previous points. This difference in major roads’ lengths between OSM and GRD can affect the results of the exposure assessment model. If the OSM data has less/more major roads compared to the GRD data, model results will be lower in both cases, since the fitting of the model will be affected.

#### 3.3.1. Road Density

Table 6 presents results of the linear model of major road density based on OSM and GRD using different buffer sizes. As shown in the results, linear model fitting results vary in the different study areas; best fits were observed in Eastern Massachusetts (R^2^ 0.92–0.94), while both Beer-Sheva (R^2^ 0.69–0.77) and Bern (R^2^ 0.80–0.89) resulted in slightly lower fits. In addition, when comparing the results in the different buffer sizes of each study area, the R^2^ does not change much for the different buffer sizes in each study area. 

Road density considers the sum of road length from both datasets inside each buffer, hence it should be affected by major road completeness. We expected to find a match between completeness (%) results (Table 3) and the road density linear model result—lower results in Eastern Massachusetts and higher results in Beer-Sheva. The results we found were affected by the difference between OSM and GRD road length. We found that high differences between OSM and GRD length (Table 5), lowered road density linear model results (Table 6). In Beer-Sheva, we found the highest difference and the lowest result in the road density linear model, while in Eastern Massachusetts, we found the lowest difference between the OSM and GRD road length, and a highest result in the road density linear model.

#### 3.3.2. Distance to Nearest Major Road

In Table 7, we present results of the exposure assessment analysis for the distance to major roads using both OSM and GRD. Higher models’ fits were found in Eastern Massachusetts (R^2^ 0.96) compared to lower fits in the Bern region (R^2^ 0.66) and Beer Sheva region (R^2^ 0.84). 

The distance to the nearest major road linear model considers the PA of roads, since we measured the distance from the building centroid to the nearest major road; if OSM roads are not as accurate as GRD, lower correlations will be found in the linear model. Hence, we expected to find a match between the distance from the nearest road linear model results (Table 7) and PA results (Table 4). We found a higher result for PA in Eastern Massachusetts (~99% completeness) and the highest linear model result of the distance to nearest roads (R^2^ 0.96). In Bern, we found PA results (~88%) and fits (R^2^ 0.66), while in Beer-Sheva the PA result was 89% with a R^2^ 0.84.

Low fitting results in the linear model could also be caused by misclassification of roads; for example, if a major road was misclassified as a minor road in OSM, the distance from the OSM major road could be higher than the distance from GRD and can lower the linear model result. 

## 4. Discussion

In this study, to the best of our knowledge, we present for the first-time environmental exposure assessment results that compare OSM and GRD. Our findings suggest that OSM road data is fairly complete and accurate (83–112%) in all study areas of Massachusetts, Bern, and the southern region of Israel. When comparing the three study areas, we found differences in the environmental assessment results: In Eastern Massachusetts, the results of both major roads models were very high (R^2^ 0.92–0.94). Results in Bern (R^2^ 0.66–0.89) and Beer-Sheva (R^2^ 0.69–0.84) were also good, yet slightly lower. We believe these results will still improve over time, since OSM data is constantly improving over time [17,18,19,24,25]. 

Previous studies found that completeness and positional accuracy is better in populated areas when compared to rural areas [17,24,25]. Our findings were consistent with these studies. We also found that in Bern city and Boston, the positional accuracy of OSM is higher than in rural areas, but when calculating completeness, we found that in Boston and Bern cities, the completeness was slightly lower (2%) compared to their corresponding region. In Beer-Sheva, we found that PA was lower and completeness was much higher compared to the southern region of Israel. These high differences between the southern region and Beer-Sheva could be due to a misclassification of the road’s type: When a minor road was classified as a major road in OSM, it could create higher results in completeness, and lower results in PA. Completeness considers the sum of road length; if more roads are considered as major roads in OSM compared to GRD, than the completeness percent will be higher. Misclassification could also lower PA results; since PA is calculated in all grids with OSM data, if GRD road is missing, than PA results in those grids will be lower.

Our study has some limitations. Although we compared exposure assessment using GRD and OSM major roads, we did not analyze the thematic accuracy of the road classification. OSM users define the type of the roads based on their knowledge or opinion [27], and thus road type misclassification could affect our results. In addition, it was shown that the number of users per area improves the quality and reliability of OSM data [11]. There is a clear difference in population density between the study areas. Eastern Massachusetts is more populated [64] compared to the Bern region [65] and southern Israel [66]. Although user data is hard to acquire, some open source tools exist that try to quantify the number of users [74]. Based on Mapbox (which tries to quantify the number of users and their edits per-country), we see that there are thousands of users in the USA (between 4000–5000) while in Israel, the number of users is much lower (400–500 users). Hence, it is possible that the difference in the number of users per area could affect our results. An additional future study should be carried out to analyze the effect of misclassification and population density on our results.

## 5. Conclusions

In summary, this study compared environmental exposure assessment results of OSM versus governmental or commercial data in three developed countries. Completeness and positional accuracy of OSM was mostly high in all study areas. When comparing the environment exposure assessment results, we saw that there is some heterogeneity in the different study areas, yet they are all relatively robust. There is a need to investigate these findings in other areas of the world, especially in undeveloped countries, where official GIS data is rare, and the dependence of VGI for exposure assessment studies is high.

## Figures and Tables

**Table 1:** Environmental assessment and epidemiological studies using OpenStreetMap (OSM) data as source information.

Reference	Study Area	Aim of the Study	OSM Data Type	Changes Made in OSM Data by the Researches	Results
54	UK	Predicted annual average daily traffic (AADT) at a national scale in minor roads, and validated the model using traffic counts and noise measurement.	Roads	No changes were made. They divide the roads into two types: Major roads (including motorway, primary, and trunk roads) and minor roads (secondary, tertiary, residential, or unclassified).	Although they found road misclassification in several areas, their methods improve noise prediction (from 0.42 to 0.72), compared to models that do not consider minor roads’ variability.
55	Israel	Estimated NO_2_ concentration using GPS-transceivers installed in vehicles.	Roads and Polygons	No changes were made. They divide the roads into five classes, and used highway segments for the analysis (motorway and trunk). Polygons data used to classify land use.	Traffic volumes were successfully used as a proxy for NO_2_. The model performed better in high traffic hours than in low traffic hours.
56	Netherlands	Estimated the spatial distribution of exposure to Q-Fever due to Coxiella burnetii spreading from goat farms. The location of a resident (based on observed outbreak data) was used as a proxy for exposure.	Buildings	No changes were made. They calculated building density as a proxy for population density.	The assessed location of the highest exposure was close to the animal market, which was the source that caused the outbreak.
57	Zurich, Switzerland	Created a high-resolution urban air pollution map, using mobile sensor measurements, installed on top of public transportation.	Roads	No changes were made. They used the pollution maps to calculate cost function to each road in Zurich to compute health–optimal routes.	They created the most accurate and timely assessment of air quality in urban areas.
58	Mexico City	Used the new data from the MAIAC AOD satellite to estimate PM_2.5_ in Mexico City using Land Use Regression (LUR) combined with the mixed effect model.	Roads	No changes were made. They calculated road density.	They developed the first high spatial and temporal model for the PM_2.5_ exposure model using satellite measurements.
59	Réunion Island, France	Studied the effect of the population’s mobility on Chikungunya (a vector-borne disease) epidemic in 2005–2006 on the Réunion Island in France.	Roads	No changes were made. They calculated road density as a proxy for population density.	Results identify human mobility as a key parameter in the spread of the epidemic. Results were validated against real epidemic data.
60	Montreal, Canada	Created a web-based route planning tool to reduce cyclists’ exposures to traffic pollution.	Roads	No changes were made. They segmented the roads on the intersection, and calculated length and average concentration of NO_2_ to each segment.	On average, the difference in exposure to NO_2_ between the shortest and alternative routes suggested in their web-tool was modest (~5%) and alone may not present a meaningful public health benefit.
61	Ulaanbaatar, Mongolia	Assessed the feasibly of LUR exposure assessment techniques, and estimated the mortality attributable to air pollution of NO_2_ and SO_2_.	Roads	Minor modifications were made based on local knowledge and location of features in the images. Roads were divided into two categories: Peace Avenue and major roads.	LUR model results of NO_2_ were between the ranges of previous studies. LUR results for SO_2_ were better than previous studies. They estimated that about 10% of deaths in 2009 were attributable to air pollution.
62	Greece	Particulate matter exposure assessment in urban areas in Greece during 2001–2010.	Roads	Data were gap filled and modified according to recent changes in road types. In addition, data was classified into four categories: Motorways, major, minor, and pedestrian.	Particular matter concentration has dropped significantly in the period of 2001–2010.
63	Portugal	Assessed the relationship between asthma hospital admission and environmental variables, including: Near surface air temperature, relative humidity, vegetation density, NO_2,_ and PM_10_. They used the Land-Use Regression (LUR) model for the assessment.	Roads	The encoding of OSM road network was not uniform, so they also used the road network provided by the Portuguese Street Authority.	Asthma hospital admissions were associated with high temperatures, low vegetation density, and high levels of NO_2_.

**Table 2:** Descriptive statistics.

	Eastern Massachusetts, USA	Boston, Eastern Massachusetts	Bern Region, Switzerland	Bern city, Switzerland	South Region, Israel	Beer-Sheva City, Israel
Total Area (km^2^)	4909.63	129.91	5970	236.86	14,511.36	117.49
Number of grids (1 km^2^)	5326	220	5841	308	14,950	149
Number of grids with road data	3604	172	1608	155	2209	58
spatial references system	NAD 1983 State Plane Massachusetts Mainland FIPS 2001		CH1903 LV03 Hotine Oblique Mercator Azimuth Center		ITM Grid	
Major road GRD types	Types 1–4		1 Klass, Autobahn, Autostr		Highway, National highway, Regional road, Local road	
Major road GRD length (km)	6592.90	809.47	1616.97	233.59	1927.08	61.41
OSM major road type	Motorway, Trunk, Primary, Secondary		Motorway, Trunk, Primary, Secondary		Motorway, Trunk, Primary *	
OSM major road length (km)	5911.68	675.39	1812.69	260.19	1995.03	99.91

* Note: In Israel, secondary roads were not selected because they are not defined locally as major roads.

**Table 3:** OSM completeness.

	Eastern Massachusetts, USA	Boston City, Massachusetts, USA	Bern Region, Switzerland	Bern City, Switzerland	South Region, Israel	Beer-Sheva City, Israel
Major GRD length (km)	6592.90	809.47	1616.97	233.59	1940.02	61.41
OSM major road length (km)	5911.68	675.39	1812.69	260.19	2005.93	99.91
Completeness (%)	89.67	83.45	112.1	111.39	103.34	162.69

**Table 4:** OSM Positional Accuracy (PA) percent.

	Eastern Massachusetts, USA	Boston	Bern Region, Switzerland	Bern City, Switzerland	South Region, Israel	Beer-Sheva, Israel
OSM 1 m buffer area (km^2^)	11.79	1.09	3.62	0.52	4.01	0.16
GRD 15 m buffer intersect area (km^2^)	11.65	1.08	3.17	0.45	3.77	0.15
GRD 20 m buffer intersect area (km^2^)	11.66	1.08	3.19	0.46	3.84	0.15
Positional accuracy (%) 15 m buffer	98.81	99.08	87.57	86.54	94.01	93.75
Positional accuracy (%) 20 m buffer	98.9	99.08	88.12	88.46	96	93.75

**Table 5:** Data used to calculate the exposure assessment.

	Eastern Massachusetts, USA	Bern Region, Switzerland	Beer-Sheva, Israel
Number of buildings used for the analysis (10% from building layer)	116,063	27,247	2278
GRD major roads selected	Types 1–4	1 Klass, Autobahn, Autostr	Highway, national highway, regional road, local road
OSM major roads selected	Motorway, Trunk, Primary, Secondary	Motorway, Trunk, Primary, Secondary	Motorway, Trunk, Primary
GRD major road total length (km)	6592.90	1616.97	52.70
OSM major road total length (km)	5911.68	1812.69	81.57
Difference between road length (The absolute difference between OSM and GRD length divided by the sum of OSM and GRD length)	0.054	0.057	0.22

**Table 6:** Road density exposure assessment linear model results. β = Beta, CI = Confidence interval, ** *p* < 0.01; *** *p* < 0.001; NS = not significant.

	Buffer Size	50 m	100 m	200 m	500 m	1000 m
		β	β	β	β	β
		(CI) ^sig^	(CI) ^sig^	(CI) ^sig^	(CI) ^sig^	(CI) ^sig^
Eastern Massachusetts, USA	(Intercept)	0	0	0	0	0
		(0–0) ***	(0–0) ***	(0–0) ***	(0–0) ***	(0–0) ***
	GRD	0.94	0.91	0.87	0.82	0.80
		(0.94–0.94) ***	(0.91–0.91) ***	(0.87–0.87) ***	(0.81–0.82) ***	(0.80–0.80) ***
	Observations	116,063	116,063	116,063	116,063	116,063
	R^2^	0.94	0.93	0.92	0.92	0.94
Bern Region, Switzerland	(Intercept)	0	0	0	0	0
		(0–0) ***	(0–0) ***	(0–0) ***	(0–0) ***	(0–0) ***
	GRD	0.94	0.95	0.96	1	1.05
		(0.93–0.94) ***	(0.94–0.95) ***	(0.95–0.96) ***	(0.99–1) ***	(1.05–1.06) ***
	Observations	27,247	27,247	27,247	27,247	27,247
	R^2^	**0.80**	**0.80**	**0.81**	**0.83**	**0.89**
Beer-Sheva, Israel	(Intercept)	0	0	0	0	0
		(0–0) ^NS^	(0–0) ^NS^	(0–0) **	(0–0) ***	(0–0) ***
	GRD	1.39	1.44	1.32	1.2	1.24
		(1.35–1.43) ***	(1.40–1.47) ***	(1.39–1.35) ***	(1.17–1.23) ***	(1.21–1.27) ***
	Observations	2278	2278	2278	2278	2278
	R^2^	**0.69**	**0.75**	**0.77**	**0.74**	**0.74**

**Table 7:** Distance from the roads exposure assessment linear model results. β = Beta, CI = Confidence interval, *** *p* < 0.001, GRD = Governmental Major Road Data, Intercept = Model intercept.

		β (CI) ^sig^
Eastern Massachusetts, USA	(Intercept)	8.63 (8.05–9.22) ***
	GRD	0.98 (0.98–0.98) ***
	Observations	116,063
	R^2^	**0.96**
Bern Region, Switzerland	(Intercept)	171.9 (161.68-182.12) ***
	GRD	0.68 (0.68–0.69) ***
	Observations	27,247
	R^2^	**0.66**
Beer-Sheva, Israel	(Intercept)	43.36 (25.99–60.73) ***
	GRD	1.05 (1.03–1.07) ***
	Observations	2278
	R^2^	**0.84**
